# Microfracture is more cost-effective than autologous chondrocyte implantation: a review of level 1 and level 2 studies with 5 year follow-up

**DOI:** 10.1007/s00167-017-4802-5

**Published:** 2017-11-11

**Authors:** Tommy Frøseth Aae, Per-Henrik Randsborg, Hilde Lurås, Asbjørn Årøen, Øystein Bjerkestrand Lian

**Affiliations:** 1 Department of Orthopedic Surgery, Kristiansund Hospital, 6518 Kristiansund, Norway; 2 Institute of Clinical Medicine, Faculty of Medicine, University of Oslo, Oslo, Norway; 3 Department of Orthopedic Surgery, Akershus University Hospital, 1478 Lørenskog, Norway; 4 Institute of Clinical Medicine, University of Oslo, Campus Ahus, 1478 Lørenskog, Norway; 5 Department of Health Services Research, Akershus University Hospital, 1478 Lørenskog, Norway; 6 Oslo Sports Trauma Research Center (OSTRC), Norwegian School of Sports Sciences, postboks 4014 Ullevål Stadion, 0806 Oslo, Norway; 7 Institute of Neuromedicine, Faculty of Medicine, Norwegian University of Science and Technology, 7491 Trondheim, Norway

**Keywords:** Microfracture, Autologous chondrocyte implantation, Articular cartilage lesion, Cost-effectiveness

## Abstract

**Purpose:**

Focal cartilage defects in the knee may have devastating effect on the knee joint, where two of the main surgical treatment options are microfracture and autologous chondrocyte implantation. Comparative studies have failed to establish which method yields the best clinical results. A cost-effectiveness analysis of microfracture and autologous chondrocyte implantation would contribute to the clinical decision process.

**Methods:**

A PubMed search identifying level I and level II studies with 5 year follow-up was performed. With the data from these studies, decision trees with associated service provision and costs connected to the two different techniques were designed. In addition to hospital costs, we included costs connected to physiotherapy following surgery. To paint a broader cost picture, we also included indirect costs to the society due to productivity loss caused by work absence.

**Results:**

Four high-quality studies, with a follow-up of 5 years, met the inclusion criteria. A total of 319 patients were included, 170 undergoing microfracture and 149 autologous chondrocyte implantation. The re-operation rate was 23 (13.5%) following microfracture, and 18 (12.1%) for autologous chondrocyte implantation. Both groups achieved substantially better clinical scores at 5 years compared to baseline. Microfracture was more cost-effective when comparing all clinical scores.

**Conclusion:**

Microfracture is associated with both lower costs and lower cost per point increase in patient reported outcome measures. There is a need of well-designed, high-quality randomized controlled trials before reliable conclusions regarding cost-effectiveness in the long run is possible.

**Level of evidence:**

III.

## Introduction

The articular cartilage in joints is composed of hyaline cartilage, with optimum load bearing and friction properties. Due to limited self-repair ability, an injury to the articular cartilage will lead to permanent damage. Focal cartilage defects (FCDs) of the knee joint may lead to severe morbidity and osteoarthritis [1], and are commonly diagnosed by magnetic resonance imaging or arthroscopy. In a retrospective study of 31,516 knee arthroscopies, Curl et al. found that 63% had cartilage injuries [2]. Årøen et al. reported that 66% of 993 knee arthroscopies had cartilage lesions, with 6% having a full thickness cartilage defect [3].

There are numerous treatment options available, where all aim to reduce pain, restore function, and minimize secondary osteoarthritis. Treatments can broadly be divided into bone-morrow stimulation techniques (microfracture), osteochondral autograft or allograft transplantation and cell-based techniques (autologous chondrocyte implantation) [4, 5]. Microfracture (MF) has gained popularity over the last few decades being a minimal invasive approach with technical simplicity and low costs [6]. In addition, there are no extra laboratory expenses or secondary surgery [7]. The MF technique described by Steadman includes debridement of the defect, before an awl is used to perforate (“microfracture”) the subchondral bone [8]. By this method, multipotent mesenchymal stem cells from the condyle are recruited to produce fibrocartilage filling of the defect. In contrast to this procedure, the most advanced cartilage procedure is the autologous chondrocyte implantation (ACI). This is a two-stage procedure, where the aim is to produce hyaline-like cartilage filling of the cartilage defect [9, 10]. First, small samples of normal cartilage tissue are harvested during a simple arthroscopy, and cultured in the laboratory. In the second operation, the cultured chondrocytes are re-implanted into the defect and mature into hyaline-like cartilage.

Short- and long-term studies have reported better function and less pain following knee cartilage surgery than prior to surgery [7, 11–13], but normal knee function is normally not achieved [6, 14, 15]. Based on cohort studies with at least 5 years of follow-up, no difference between the various surgical methods in regard to clinical scores, failure rates, and secondary surgeries has been found [16–19]. One study reported that MF and osteochondral autograft transplantation are equally cost-effective treatment options in a 10 year perspective [20]. Previous published studies comparing costs following MF and ACI have not analysed only high evidence studies with a minimum follow-up of 5 years, nor taken into account all the costs related to the procedure. Most notably, the costs of physiotherapy following the procedures are sparse [21, 22], whereas indirect costs to the society related to sick leave are almost absent [23].

Given the increased focus on health care efficiency and the high prevalence of FCDs on the distal femur, one should try to identify the most cost-effective treatment option to contribute to the clinical decision process for these troublesome injuries.

Previous published cost-effectiveness analysis is based on short term follow-up only. The purpose of the current study is to compare costs after 5 years between MF and ACI, based on pre-existing level 1 and level 2 studies.

## Materials and methods

Miller et al. performed a cost-effectiveness analysis of cartilage injuries comparing microfracture and osteochondral autograft transplantation [20]. In the current study, we extent their method by also including costs for physiotherapy and indirect costs to society due to sick leave. A literature search was carried out in January 2017 using the database of PubMed, for clinical trials phase I and II studies comparing MF and ACI for the treatment of FCDs in the distal femur with a minimum 5 years of follow-up. Using the keywords “microfracture”, “autologous chondrocyte implantation”, “cartilage repair”, “cartilage lesions”, “mosaicplasty”, “osteochondral transfer and transplantation”, “osteochondral autograft” and “osteoarticular transfer system”, only publications in English were included. Articles with reported evidence level I and II were included. Studies regarding the paediatric and adolescent population were excluded (as these focus on osteochondritis dissecans). As long as they met inclusion criteria, studies comparing other cartilage procedures were included.

According to the standard methods for economic evaluation of health care programs, decision trees following MF and ACI as previously described by Drummond et al. were constructed [24]. Terminal endpoints were either success or failure, where the latter was defined as pain or loss of function which required revision surgery. Based on the decision tree and clinical experience regarding service provision in the two different alternatives, treatment paths were constructed for MF and ACI, respectively. The cost data were taken from a local orthopedic hospital in Norway, and verified via personal communication with other orthopedic hospitals in the country. Direct costs including physiotherapy was first calculated, and second indirect costs related to sick leave was included. Hospital costs (unit prices) were based on a cost-per-patient calculation model, which is an established standard for calculating patient-level costs in hospitals [25] (Table 1).

**Table 1:** Total cost primary surgery per patient

Variable	Unit price (€)	Cost (units)	
		MF	ACI
Initial consult	95	95 (1)	95 (1)
Surgery and material		1749	3498
Cell culture	4050		4050
Outpatient follow-up visit	35	70 (2)	70 (2)
Hospital stay (each night)	620	620 (1)	1860 (3)
Physiotherapy	30	720 (24)	1440 (48)
Direct costs		3254	11,013
Indirect costs (absent from work)	215	1075 (5)	3225 (15)
Total costs		4329	14,238

All costs in Euros (€) per patient

*MF* microfracture, *ACI* autologous chondrocyte implantation, *N/A* not applicable

The costs of revision surgery were calculated for the specific procedures (diagnostic arthroscopy, MF, mosaicplasty, ACI, high tibial osteotomy, and total knee arthroplasty), and included costs of a magnetic resonance imaging and a return visit. For the costs of one overnight stay in an orthopedic ward, a Norwegian estimate is €620 [26]. The length of hospital stays following MF and ACI varies, where stays up to 4 days have been reported [23, 27]. In this study, lengths of stay are set to 1 day for MF and 3 days for ACI which corresponds to both clinical practice and the current literature [28] (Table 2). In regard to revision surgery, we assumed hospital stays of 1 day following diagnostic arthroscopy and mosaicplasty, and 3 days for high tibial osteotomy and total knee arthroplasty.

**Table 2:** Total cost revision surgery per patient

Variable	Unit price(€)	Cost (units)					
		DA	MF	MOS	ACI	HTO	TKA
Cost revision surgery							
Return visit	95	95 (1)	95 (1)	95 (1)	95 (1)	95 (1)	95 (1)
MRI	198	198 (1)	198 (1)	198 (1)	198 (1)	198 (1)	198 (1)
Revision surgery and material		1749	1749	3098	3498	8030	10,563
Cell culture	4050				4050		
Outpatient follow up	35	70 (2)	70 (2)	70 (2)	70 (2)	70 (2)	70 (2)
Hospital stay	620	620 (1)	620 (1)	620 (1)	1860 (3)	1860 (3)	1860 (3)
Physiotherapy	30	720 (24)	720 (24)	720 (24)	1440 (48)	1440 (48)	1440 (48)
Direct cost		3452	3452	4801	11,211	11,693	14,226
Indirect cost (absent from work)	215	1075 (5)	1075 (5)	1075 (5)	3225 (15)	3225 (15)	3225 (15)
Total cost revision surgery		4527	4527	5876	14,436	14,918	17,451

All costs in Euros (€) per patient

*MRI* magnetic resonance imaging, *DA* diagnostic arthroscopy, *MF* microfracture, *ACI* autologous chondrocyte implantation, *MOS* mosaicplasty, *HTO* high tibial osteotomy, *TKA* total knee arthroplasty

In regard to postoperative physiotherapy, there is no consensus regarding frequency or duration. Our assumptions are based on clinical experience and personal communications. After ACI, Brittberg recommends physiotherapy twice weekly for 24 weeks (personal communication), while Robert LaPrade at Steadman’s clinic recommends physiotherapy twice weekly for 12 weeks following MF (personal communication). When estimating the costs of physiotherapy following revision surgery, we assumed physiotherapy twice weekly for 12 weeks after diagnostic arthroscopy, MF and mosaicplasty, and twice weekly for 24 weeks for ACI, high tibial osteotomy and total knee arthroplasty. The unit cost of one session physiotherapy is €30 (The Norwegian Physiotherapist Association). No brace was included in the costs.

A human capital approach (HCA) was employed to calculate indirect costs (productivity loss). In HCA, the loss to the society is estimated from the income normally earned by the patients [29]. The idea is that the employees’ wages provide an estimate of the value their labour contributes to the economy, and labour that is lost due to sick leave is assumed to reduce the society’s total productivity accordingly. A total of 5 days off work is expected following MF surgery, and 15 days following ACI [23]. Based on data from Statistics Norway (2016), a fulltime employee (both genders) aged 30–34 years earns €4667 per month, or €215 for each day absent from work [30]. This age range corresponds to the average age of the patient population. Assessing the indirect costs (sick leave) following revision surgery, we assumed 5 day off work for diagnostic arthroscopy, MF and mosaicplasty, and 15 day off work for ACI, high tibial osteotomy and total knee arthroplasty.

Total costs at 5 years are calculated by summing primary costs and costs for revision surgery.

By comparing total costs and the weighted average of the reported outcome measures, we calculated the costs related to a 1-point increase in each of the reported PROM values following MF and ACI. All costs were converted to 2017 Euros based on the Norwegian consumer price index.

A sensitivity analysis was performed to calculate alternative values to see how sensitive the end result is for the choice of value on different variables. Guidelines often discount costs at a 3% annual rate, considered to start at 5 years [31]. Since our study has a follow-up of 5 years, the costs were not discounted.

## Results

**Fig. 1:**
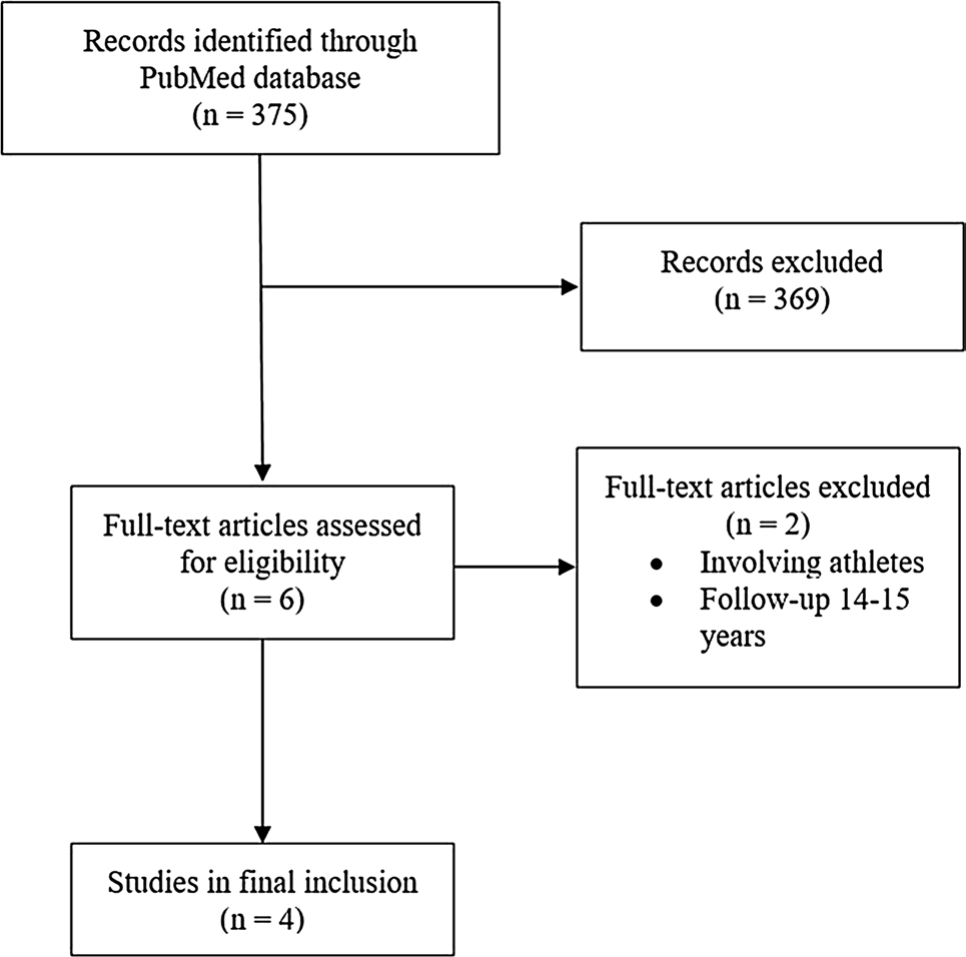
Flow diagram of article selection included in the study

Six studies were identified [16–19, 32, 33] (Fig. 1), corresponding to three systematic reviews [11, 34, 35]. Three studies compared MF with ACI using periosteum [16, 18, 32], two compared MF with scaffold ACI [17, 33], whereas one compared MF with characterized chondrocyte implantation (CCI) [19]. One study involving high level athletes did not report failures, and was excluded [33]. One author had published results both after 5 and 14–15 years [16, 32], but only the 5 year results were included. Hence, 4 articles with 319 patients (208 males, 65%) formed the basis for comparison of clinical scores schemes, failure rates and revision surgeries [17–19, 32] (Table 3).

**Table 3:** Summary of the included articles

Level	References	Technique	Patients	PROMs
I	Knutsen et al. [32]	MF—P-ACI	40–40	VAS, Lysholm, Tegner, SF-36
II	Lim et al. [18]	MF—P-ACI	29–18	Lyshom, Tegner, HSS Knee score
II	Kon et al. [17]	MF—scaffold ACI	40–40	IKDC, Tegner
I	Vanlauwe et al. [19]	MF—CCI	61–51	KOOS

*MF* microfracture, *P-ACI* autologous chondrocyte implantation using periosteum, *PROMs* patient reported outcome measures, *VAS* visual analogoue scale, *SF-36* short form 36, *HSS* hospital for special surgery, *KOOS* Knee Injury and Osteoarthritis Outcome Score, *IKDC* International Knee Documentation Committee, *Ref* reference number

**Fig. 2:**
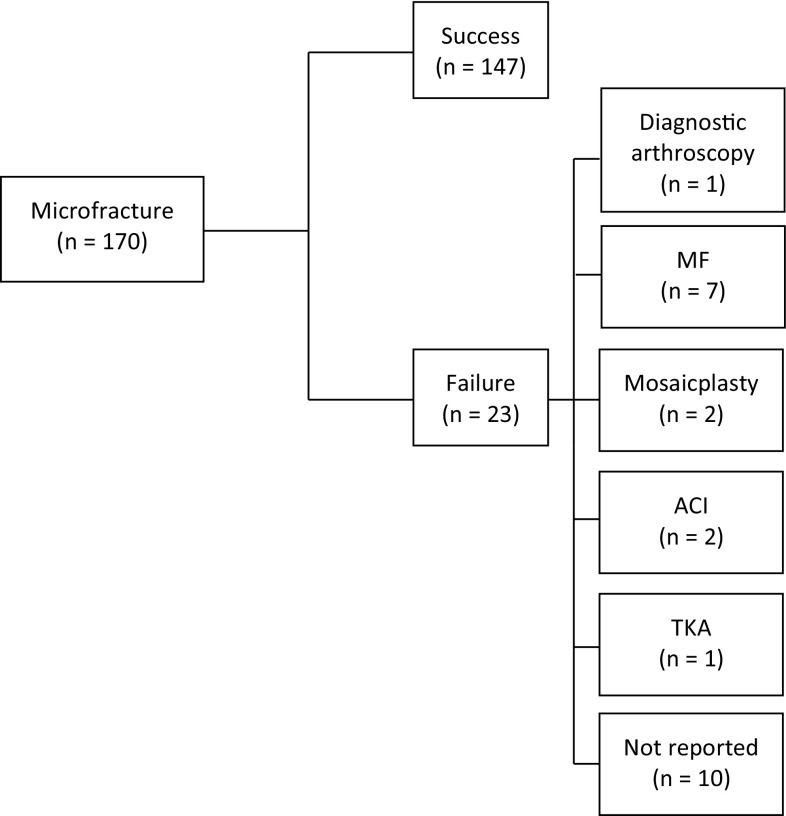
Microfracture decision tree. *n* number of patients, *MF* microfracture, *ACI* autologous chondrocyte implantation, *TKA* total knee arthroplasty

**Fig. 3:**
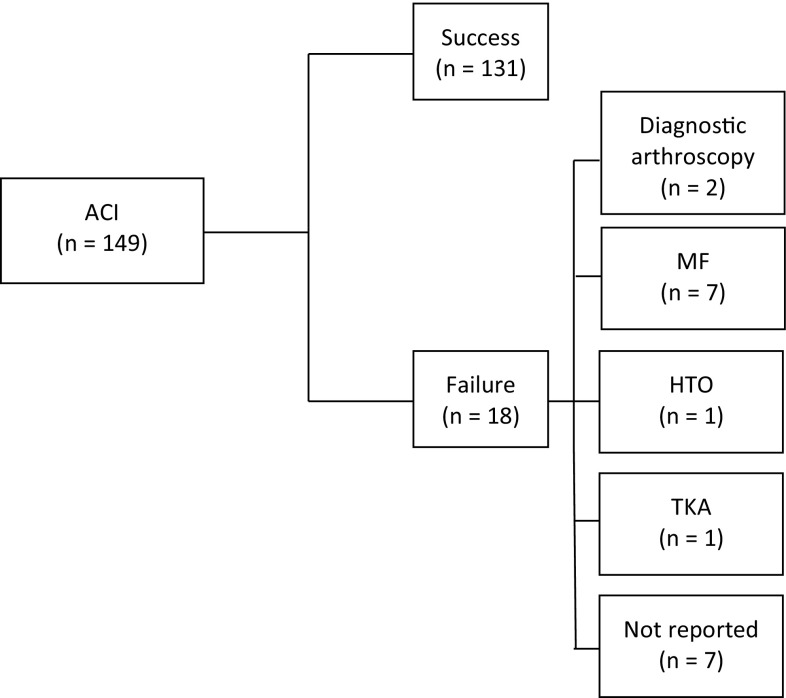
Autologous chondrocyte implantation decision tree. *n* number of patients, *MF* microfracture, *HTO* high tibial osteotomy, *TKA* total knee arthroplasty

170 patients underwent MF, and 149 ACI. Patients in the two groups were 32.1 (MF) and 33.1 (ACI) years, with lesion sizes 2.5 cm^2^ (MF) and 3.2 cm^2^ (ACI). Based on the decision trees, 147 (86.5%) in the MF group, and 131 (87.9%) in the ACI group achieved success at 5 years (Figs. 2, 3).

One study did not specify treatment failure [32]. One study reported re-intervention rates, but did not specify the revision procedure [19]. For our cost analysis, we assumed the non-specific revisions as diagnostic arthroscopies (ten in the MF group and seven in the ACI group).

MF had direct costs of €3254 at baseline (Table 1), rising to €3892 at 5 years (Table 4), while ACI was €11,013 at baseline, increasing to €11,558 at 5 years. When we included productivity loss due to sick leave, MF had total initial costs of €4329 rising to €5150 at 5 years. For ACI, the total costs at baseline and at 5 years were €14,238 and €14,941, respectively. Total costs connected to revision surgery were slightly higher in the MF group (€821) compared to ACI (€703) (Table 4).

**Table 4:** Total costs at 5 years

		MF (patients)	ACI (patients)
Direct costs			
Primary surgery		553,180 (170)	1,640,937 (149)
Revision surgery			
	Unit price (€)		
DA	3452	3452 (1)	6904 (2)
MF	3452	24,164 (7)	24,164 (7)
MOS	4801	9602 (2)	N/A
ACI	11,211	22,422 (2)	N/A
HTO	11,693	N/A	11,693 (1)
TKA	14,226	14,226 (1)	14,226 (1)
Not reported	3452	34,520 (10)	24,164 (7)
Direct costs		661,566 (170)	1,722,088 (149)
Direct costs per patient		3892	11,588
Total costs			
Primary surgery		735,930 (170)	2,121,462 (149)
Revision surgery			
	Unit price (€)		
DA	4527	4,527 (1)	9054 (2)
MF	4527	31,689 (7)	31,689 (7)
MOS	5876	11,752 (2)	N/A
ACI	14,436	28,872 (2)	N/A
HTO	14,918	N/A	14,918 (1)
TKA	17,451	17,451 (1)	17,451 (1)
Not reported	4527	45,270 (10)	31,689 (7)
Total costs revision surgery		139,561 (170)	104,801 (149)
Revision surgery costs per patient		821 (23)	703 (18)
Total costs		875,491 (170)	2,226,263 (149)
Total costs per patient		5150	14,941

The non-specific revisions listed as not reported were assumed as diagnostic arthroscopy in the cost analysis. The cost calculations after primary surgery is based on Table 1

All costs in Euros (€)

*DA* diagnostic arthroscopy, *MF* microfracture, *MOS* mosaicplasty, *ACI* autologous chondrocyte implantation, *HTO* high tibial osteotomy, *TKA* total knee arthroplasty, *N/A* not applicable

Different validated patient reported outcome measures (PROMs) were used. The Tegner score was used in three studies [17, 18, 32], the Lysholm score was reported in two [18, 32], whereas the visual analogue scale (VAS) [32], Short Form 36 (SF-36) [32], Hospital for Special Surgery (HSS) [18], the Knee Injury and Osteoarthritis Outcome Score (KOOS) [19] and the International Knee Documentation Committee (IKDC) [17] were reported in one study each. Comparing the weighted average of the preoperative PROMs with the weighted average of the PROMs after 5 years, all reported statistically clinical improvement for both MF and ACI [34, 36]. Based on the weighted average of the PROMs, a comparative cost-effectiveness analysis was carried out given a 1-point increase on each of the reported clinical scores for total costs at 5 years. For all measures, a 1-point increase in clinical scores had lower costs for MF than for ACI at 5 years (Table 5).

**Table 5:** Cost per 1-point improvement in the patient reported outcome measures for total cost at 5 years

PROM	PROM difference	Costs per point
	Baseline—5 years	Improvement per patient (€)
VAS		
MF	29	178
ACI	28	534
Lysholm		
MF	26	198
ACI	19	786
Tegner		
MF	1.8	2861
ACI	2.8	5336
SF-36		
MF	10	515
ACI	7	2134
HSS		
MF	9.4	548
ACI	10.0	1494
KOOS		
MF	14.1	365
ACI	21.2	705
IKDC		
MF	30	172
ACI	42	356

All costs in Euros (€)

*PROM* patient reported outcome measure, *MF* microfracture, *ACI* autologous chondrocyte implantation, *VAS* visual analogue scale, *SF-36* short form 36, *HSS* hospital for special surgery, *KOOS* Knee Injury and Osteoarthritis Outcome Score, *IKDC* International Knee Documentation Committee

The sensitivity analysis showed that a 66% reduction in the total costs following ACI or a 190% increase in the total costs of MF led to equivalent total costs at 5 years. Comparing only primary direct costs, a reduction in costs of 70% after ACI, or a 239% increase in costs after MF would lead to equivalent costs at baseline. Assuming identical costs for hospital stay, physiotherapy and sick leave after the primary surgery, an increase in costs of 69% following MF and a decrease in costs of 41% after ACI would lead to identical total costs after the primary surgery.

## Discussion

The most important finding of the present study was that MF is more cost-effective than ACI for the treatment of FCDs in the distal femur with 5 year follow-up. The main difference in total costs is related to the primary surgery, where MF is less expensive than ACI. Costs following revision surgery are however lower in the ACI group, respectively, €703 (ACI) and €821 (MF) per patient.

The included studies have demonstrated that the clinical scores are statistically significantly better at 5 years compared to pre-surgery for both methods. Not all studies reported variances or standard deviations, so we were unable to calculate a precise *p* value that demonstrates that the difference in cost-effectiveness is statistically significant. However, given the large differences in costs per point improvement between MF and ACI, it is unlikely that our findings are purely coincidental.

In a recent study comparing MF, osteochondral autograft transplantation and ACI, Schrock et al. reported that MF was the most cost-effective treatment option for chondral lesions in the knee, confirming our findings [37]. In contrast, Mistry et al. reported ACI to be cost-effective compared to MF [22]. When calculating costs, Mistry et al. assumed ACI to be performed as outpatient surgery with a total of six outpatient follow-ups, while MF was assumed to be inpatient surgery. Because ACI is a far more invasive procedure than MF, we assumed 3 days of hospitalization and two outpatient follow-ups after ACI, and 1 day of hospitalization following MF. This is the most important reason why our results differ to Mistry et al. In addition, our study adds costs related to sick leave.

There is a wide variation between surgeons in relation with indication for surgery, preferred surgical technique, postoperative physiotherapy, and outcome assessment [38]. Some have suggested that MF should be performed as first procedure in FCDs on the femur due to the simplicity of the procedure and the associated low costs [6, 7, 39]. MF has, therefore, become the gold standard to which other methods have been compared in clinical trials [27, 40].

The effect of the cartilage lesion size on symptoms is poorly investigated. Some authors recommend ACI for cartilage lesions larger than 4 cm^2^ [7, 41], but the literature is unclear on lesions ranging from 2 to 4 cm^2^ [42]. The average lesion sizes in our study were slightly different between the groups (2.5 cm^2^ for MF and 3.2 cm^2^ for ACI), which probably do not affect our result. Recent research on anterior cruciate ligament reconstruction in combination with articular cartilage injury has found the effect of the lesion size to be minor [43].

Kon et al. compared MF with scaffold ACI and found a small clinical benefit in favour of ACI after 5 years [17]. Knutsen et al. reported no clinical significant differences comparing MF and ACI after 14–15 years [16]. Radiological early signs of osteoarthritis were found in 48% in the MF group and 57% in the ACI group. In a long-term perspective, this could affect the cost-effectiveness of these two methods.

There are technological advances both within MF and ACI. Nanofracture, scaffolds, and autologous matrix-induced chondrogenesis are gaining popularity, and may give a different clinical and cost picture of microfracture derived procedures. Scaffold may induce significantly higher costs when comparing MF with other cartilage procedures. Published papers on ACI are mainly based on first generation procedures, but second and third generation ACI have now been implemented both in clinical trials and practice. However, long-term results are not yet available [44]. Besides, the use of characterized chondrocytes implantation may yield different results than ACI. A third factor is that ACI is performed as a two-stage procedure. The development of one-stage procedures may yield different health economic effects than traditional ACI, and would probably lower the costs substantially [23].

This study has limitations. Studies with evidence levels 1 and 2 comparing MF and ACI with a minimum follow-up at 5 years are few, leading to relatively small study populations. This may lead to bias and affect the results published in this article. Yet, to this date, these are the only high-quality studies with 5 year follow-up.

Another limitation is the fact that the MF group had slightly smaller lesions and, therefore, might represent patients which are more responsive to physiotherapy after surgery. Supervised physiotherapy has also been shown to be effective together with debridement of the lesion [45], and our study cannot determine which method yield better clinical results.

Knutsen et al. published the SF-36 and Tegner score only for the success patients, and not for the failures [32]. This may lead to an overestimation of these scores, as we must assume that failures would have lower scores than the successes.

Physiotherapy before surgery and costs related to independent training are not included in our calculations because we assume them to be similar for the two groups. A wide range of postoperative physiotherapy protocols after MF and ACI exists. Some permit weight-bearing, while others use continuous passive motion (CPM) [13, 45–48]. These differences may affect the cost calculations.

Ten patients in the MF group and seven in the ACI group were re-operated, and the procedure was assumed to be diagnostic arthroscopies. If we assumed another re-operation procedure, this would affect the cost estimates. When estimating the costs related to hospital stay, work absence, and physiotherapy after revision surgery, we used the cost estimates from primary surgery, which may be an underestimation.

In regard to capital costs, account investments and orthopedic skills were not taken into account, i.e., we have assumed that hospitals can switch between MF and ACI, which in practice is not the case.

The unit prices employed in our calculations is extracted from a local orthopedic hospital, and confirmed with other orthopedic hospitals in Norway, which may limit the transferability of the study. An international cost analysis is difficult to perform because different countries face different institutional and financial constraints, including different unit prices. On the other hand, their assumption regarding service provision related to surgery, postoperative physiotherapy and sick leave are comparable to other studies, thereby giving a certain degree of transferability globally [22, 23, 37].

The results are based on 5 year follow-up. In light of cartilage pathologies, this may be sparse. However, failures usually occur within 2–3 years after the initial surgery [49, 50], so our timeline seems sufficient to capture failures. Furthermore, none of the included studies compared surgical treatment with conservatively treatment, so the true effect of surgery is in fact not known [35, 51]. High-quality studies with follow-up exceeding 5–10 years with a conservative control group are needed to be able to draw conclusions on this painful and morbid disease. Treatment of FCDs is expensive for the society, and our study may contribute to the decision process in clinical practice. This study has a broader perspective than previous cost analyses and should be of particular interest for orthopedic surgeons of this particular knee injury.

## Conclusion

There is evidence for the benefits of cartilage repair surgery using MF and ACI based on the 5 year results published when evaluating health costs related to the procedures. The MF procedure is more cost-effective than ACI based on published 5 year results, but there is a need of well-designed, high-quality randomized controlled trials with long-term results before safe conclusions can be made.

## Abbreviations

ACI: Autologous chondrocyte implantation
AMIC: Autologous matrix-induced chondrogenesis
CCI: Characterized chondrocyte implantation
CPM: Continuous passive motion
FCD: Focal cartilage defect
HCA: Human capital approach
IKDC: International Knee Documentation Committee
KOOS: Knee injury and Osteoarthritis Outcome Score
MF: Microfracture
PROMs: Patient reported outcome measures
